# Advancing Women’s Global Health Leadership: Lessons from a Tripartite Model

**DOI:** 10.5334/aogh.5285

**Published:** 2026-07-08

**Authors:** Kamla Ross McGregor, Erica Di Ruggiero, Maylene Shung-King, Faith Yego

**Affiliations:** 1 Centre for Global Health, Dalla Lana School of Public Health, University of Toronto, Toronto, Canada; 2 Social and Behavioural Sciences Division, Centre for Global Health, Dalla Lana School of Public Health, University of Toronto, Toronto, Canada; 3 Health Policy Systems Division, School of Public Health, University of Cape Town, Cape Town, South Africa; 4 Department of Health Policy Management and Human Nutrition, School of Public Health, Moi University, Eldoret, Kenya

**Keywords:** women in global health, African women leaders, capacity building, gender equity, health systems strengthening in Kenya and South Africa, north and south academic collaboration, Canada

## Abstract

*Background:* Women in the Global South remain underrepresented in high-level leadership positions in global health. Three academic institutions developed the Women in Global Health Leadership Fellowship (WGHLF) to build and strengthen the capacity of emerging women leaders in global health in Kenya and South Africa.

*Objective:* The purpose of this study was to evaluate how well the program met its intended outcomes in its first two years of operation.

*Methods:* Twenty-eight participants (12 in 2024 and 16 in 2025) were evaluated. Participants included early- to mid-career women working in health policy, practice, and academia. We analyzed pre- and post-assessment data, mid-year surveys, and focus group responses.

*Results:* Participants reported considerable knowledge and skill gains in global health policy, gender equity, leadership, and mentorship as well as increased confidence in their ability to succeed in higher-level leadership roles. Participants also noted an increased ability to lead gender equity projects at their workplace. Reported program strengths include high participant engagement, collaborative teaching approaches, growth in participant self-awareness, and the practical application of knowledge through their required leadership projects.

*Conclusions:* The WGHLF improved participants’ confidence in advancing gender equity practices in local, regional, and global health. This type of fellowship training, offered in partnership between institutions in the Global North and Global South, can serve as a collaborative model for others seeking ways to help qualified women prepare for higher-level leadership positions, which are crucially needed to advance gender equity and local health systems.

## Background

Women remain underrepresented in leadership globally, particularly in low- and middle-income countries (LMICs) and including in global public health [1–4]. While 70% of the global public health workforce are women, women occupy only 25% of senior leadership positions [3]. Although recent data show gains in women’s representation in senior roles within global health nonprofit organizations, these improvements are uneven: only 15% of board chairs in LMICs are women and 87% of these organizations are headquartered in high-income countries (HICs) [5]. Notably, most research on assessing the presence of women in global health leadership takes place in high-income settings [3, 6].

Limited research and inadequate gender representation in LMICs are problematic given their unique social, cultural, and political contexts [6, 7]. Recent sociopolitical events have led to a pushback against gender equity and sexual and reproductive health rights [8–10]. These harmful narratives have resulted in reduced access to health care, HIV/AIDS prevention and treatment, and family planning services, programs that impact women’s health [11]. There is a risk that leaders who adhere to conservative norms will use this moment to oppose gender equity initiatives and women in leadership [12]. Together, these contextual factors underscore the urgent need for institutions to address gender inequities and advance women’s leadership pathways at the highest levels in global health.

Addressing these gaps requires innovative and contextually relevant approaches to support women’s leadership [6, 7]. In response to these gaps, several programs have been developed to strengthen women’s leadership capacity. One such program is the Women in Global Health Leadership Fellowship (WGHLF), which was designed to build the leadership capacity of emerging women leaders in selected LMICs. Co-created by the Dalla Lana School of Public Health (DLSPH) at the University of Toronto (UofT), the School of Public Health at Moi University (Moi) in Kenya, and the School of Public Health at the University of Cape Town (UCT), the fellowship integrates knowledge in global health policy and gender equity, leadership and mentorship training. To ensure that leadership programs such as the WGHLF are effective, evaluations need to be conducted in real time considering program design elements (e.g., which intervention components have which effect?), program viability (e.g., how useful is the program for its intended audiences?), and program impacts (e.g., how effective is the program in achieving its intended outcomes?). Routine evaluation allows for mid-course corrections while maximizing programmatic strengths [8].

This paper outlines the partnership model, participant demographics, program components, early evaluation findings, and lessons for institutions seeking to spearhead similar initiatives to advance women’s leadership in LMICs.

### Tri-Academic partnership initiation

**Figure 1:**
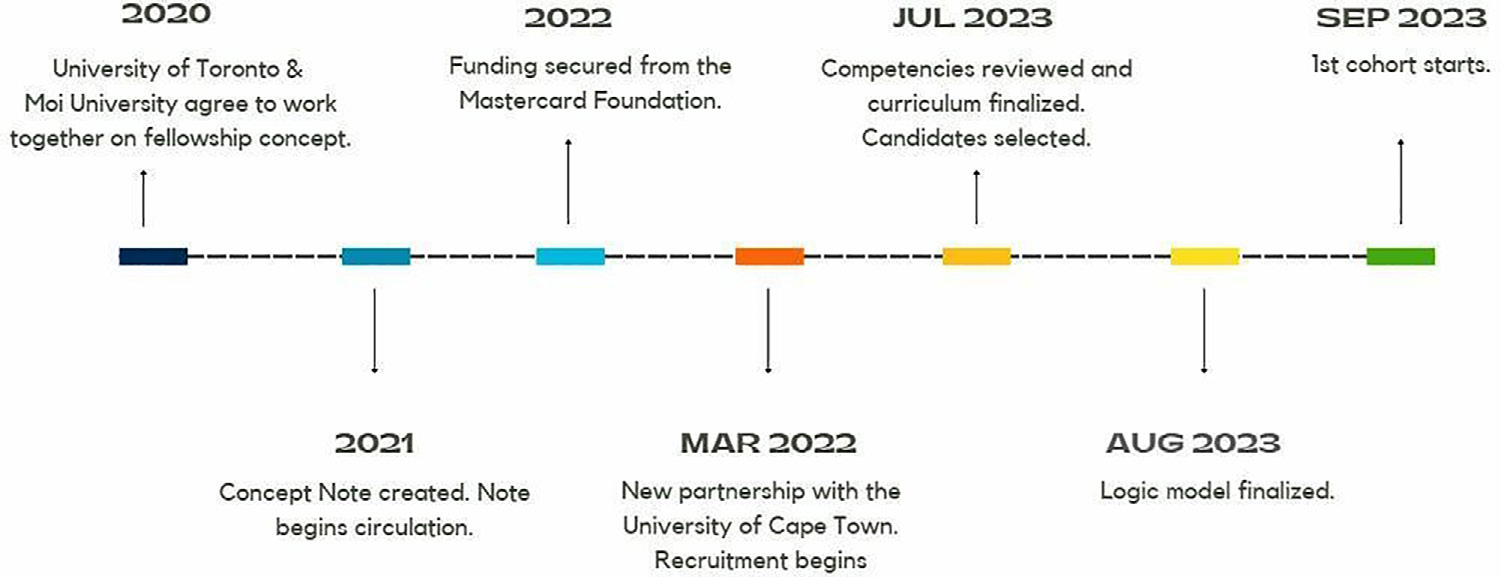
Program development timeline.

The partnership began in 2020 when faculty at Moi’s School of Public Health and DLSPH’s Centre for Global Health came together to address a shared interest—advancing women’s leadership capacity in LMICs. These institutions had collaborated since 2017 guided by a Memorandum of Understanding focused on collaborative health systems research, education, and trainee exchanges. A concept note was co-developed by both schools in 2021, and by 2022, the fellowship secured funding through the Mastercard Foundation’s Africa Health Collaborative (AHC), a network of nine higher education institutions established to strengthen Africa’s health workforce education and health systems [13]. Through the AHC, leadership at the School of Public Health at UCT became aware of the fellowship and expressed interest in joining the partnership. This new development provided an opportunity for UofT and Moi to further refine the fellowship concept with UCT faculty. Out of this shared effort, a new tri-partnership was created with space for both North-to-South and South-to-South collaboration. Based on needs, the partners agreed to target early- to mid-career women in public health, policy, teaching, and/or research roles, focusing on African nationals residing in Kenya or South Africa. Finally, the program was launched in September 2023 (see Figure 1). The creation of the tri-partnership bears insight into how shared values, complementary expertise along with institutional and donor support, were built over a three-year period to lay the foundation for launching the fellowship.

### Program description

The WGHLF program aims to equip emerging women leaders with the necessary knowledge, skills, and support to challenge institutional and systemic barriers to gender equity and develop actionable solutions in global health systems. The program offers deep personalized learning to a carefully selected set of participants to maximize peer-to-peer learning and mentorship outcomes.

### Competencies

Informed by the literature, the program is anchored in four broad competencies:

1. *Gender equity and intersectionality (an overarching competency):* The ability to employ an intersectional and/or gender equity lens to health systems and/or health systems policy.
2. *Global health policy:* The ability to contribute to policy development, communication, and advocacy to address the political environment and organizational culture of health institutions.
3. *Leadership:* The ability to create a vision that empowers others, fosters collaboration, guides decision-making, and supports diverse team members to recognize and challenge gender inequity within their organization.
4. *Mentorship:* The ability to enter a mentoring relationship to enable independent thinking and relational leadership capacities.

The 12-month program is implemented in **three phases**:

- *Phase 1—Foundations (September–December):* 12 weekly 2-hour online sessions that cover competencies 1 and 2.
- *Phase 2—Leadership and mentorship (February–April):* 4 weekly 2-hour online sessions and 5 days in-person training (either in Kenya or South Africa). Participants start mentoring and pitch leadership project ideas.
- *Phase 3—Putting it all together (May–August):* 12 weekly 2-hour online sessions where participants reinforce knowledge in all competencies and present their final leadership project plan.

The fellows spend a minimum of 98 contact hours with faculty, staff, mentors, and a career coach, not including time spent on readings, online discussion forum, and leadership project planning and execution.

### Recruitment

Recruitment is a shared responsibility. UofT prepares communication materials for review by partners, while Moi and UCT conduct in-country targeted recruitment. Moi has promoted through other university departments, and affiliated institutions such as the Moi Teaching and Referral Hospital and other external partners in Eldoret and across Kenya. In South Africa, UCT targets academic, civil society, and government health departments in the Western Cape region.

Following targeted program promotion in July 2023, the program received 27 applications, of which 12 were selected for the initial cohort. In July 2024, the program received 68 applications, of which 16 were selected (See participant demographics in Table 1). The jump in applications in year 2 is attributed to word-of-mouth referrals from the inaugural cohort and more concerted recruitment efforts by in-country partners.

**Table 1:** Participant demographics.

CHARACTERISTICS	YEAR 1 2023/2024	YEAR 2 2024/2025
**Gender** Women	12 (100%)	16 (100%)
**Country of residence** Kenya South Africa	6 (50%) 6 (50%)	8 (50%) 8 (50%)
**Career stage** 5–10 years 10–15 years Over 15 years	4 (33%) 5 (42%) 3 (25%)	4 (25%) 11 (69%) 1 (6%)
**Level of education** Bachelors Masters Post graduate diploma PhD	5 (42%) 4 (33%) 1 (8%) 2 (27%)	1 (6%) 12 (75%) 0 3 (19%)
**Employment setting** Academia Government health care Government admin./policy Private health care Nongovernmental org. Research center	3 (25%) 4 (33%) 4 (33%) 0 0 1 (8%)	5 (31%) 6 (38%) 1 (6%) 1 (6%) 2 (13%) 1 (6%)

### Attendance and completion policy

Participants must attend 80% of all online sessions in each phase, complete the in-person training, participate in all online discussion questions, prepare a leadership project plan, and show evidence of progress on the project to complete the fellowship.

### Co-Facilitation

Faculty from all three institutions work together in pairs to co-facilitate foundational topics. Supplemental sessions are facilitated by guest experts and the program coordinator.

### Stipend

Participants receive a nominal stipend to support childcare, eldercare, travel, and learning resources, among others, required during program participation. The stipend acknowledges the unpaid work to sustain their families and communities that many career women do to achieve their goals.

### Mentorship

Senior global health leaders from South Africa, Kenya, Ghana, and Canada serve as mentors. The program’s mentorship model is relational and nonhierarchical, creating space for independent thinking, critical reflection, and personal and professional growth. Mentors and mentees participate in three mandatory 2-hour online group training sessions. These sessions focus on fostering inquiry and dialogue, enabling mentors to act as facilitators rather than directors of knowledge. Mentors work one-on-one with a mentee and meet a minimum of once a month from phase 2 until the end of the fellowship.

### Coaching

Participants attend two group sessions with a leadership coach. The coach reviews the general meaning of individual leadership assessment results and answers questions. In year 2, a 1-hour individual session with the coach was added to dig deeper into their assessments’ meanings and connect to fellowship learnings.

### Leadership project

Participants pitch a project idea during the in-person week. Participants are then asked to get support or buy-in from their managers before starting. In phase 3, participants are given time during sessions to workshop their draft project plan.

Twenty-eight learners (12 in year 1 and 16 in year 2) successfully launched 27^1^ projects. Some projects included: “Applying a gender lens to a new masters course in global surgical systems,” “Improving patient experience at a psychiatric hospital through staff training on gender identity,” “Creating a gender-based violence unit at local hospital,” [14] “Evaluating and Re-Introducing a Workplace Breastfeeding Policy,” and “Starting a men’s health clinic at a local hospital” [15, 16].

### Research questions and methods

The evaluation sought to answer, “How well is the program meeting its intended short-term outcomes?” The analysis was guided by the following **research questions:**

1. How does participation in the WGHLF influence the leadership development, gender equity awareness, and advocacy skills of early- to mid-career women in global health in Kenya and South Africa?
2. How well is the program meeting its intended outcomes for participants and mentors?
3. What are the implications for similar initiatives looking to advance women’s leadership in global health, specifically from LMICs?

Evaluation questions were aligned with program outcomes, which were developed through a pre-launch logic model mapping exercise. This exercise used the program’s objectives and local contexts to clarify needed resource inputs and activities, which then determined the most logical program outcomes (see logic model in supplemental section).

The evaluation design followed Patton’s utilization-focused evaluation principles. Specifically, it emphasized the co-developing of questions by program partners, identifying methods that generate credible results in support of intended uses, and organizing data to facilitate interpretation and analysis by intended users (program partners and ultimately the funder). These principles guided recommendations to inform future program enhancements and improvements. This approach was also used to maximize the evaluation’s utility for interorganizational learning, and future scaling and sustainability [17]. This led to the use of mixed methods for the program’s evaluation (see Table 2). A mixed methods design deploys both quantitative and qualitative approaches, providing an opportunity to triangulate findings across multiple data sources, confirming points of convergence while highlighting points of divergence. Evaluations included a pre-assessment of participants’ baseline knowledge and confidence, phase 1 to 3 surveys, staff notes from individual conversations, a post-assessment to measure changes to baseline, and an end-of-program focus group. In keeping with the evaluation questions and program outcomes, new evaluation tools were developed and reviewed by all partners, and pilot-tested internally.

**Table 2:** Evaluation methods.

PHASE	EVALUATION METHOD	WHEN
Pre-start	Pre-assessment survey	Early September
Phase 1	Verbal group check-in during an online session Satisfaction survey Focus group	Mid November December
Phase 2	Notes from group observations and from conversations with individual learners during in person training Satisfaction survey	March April
Phase 3	Mid-phase survey and check-in phone calls -> (Completed by mentors) Group check in -> (completed by mentors) End-of-program satisfaction survey -> (completed by mentors) End-of-program focus group	Early June Early August
End	Post-assessment survey	End of August

The online assessments used four Likert scales measuring levels of agreement, knowledge, satisfaction, and usefulness. Positive responses for each scale were combined (top two box scoring) to summarize descriptive results for reporting purposes; for example, for agreement, “strongly agree” and “agree” responses were aggregated. Given the small sample size (Y1 = 12; Y2 = 16), a descriptive statistical approach was selected as the most appropriate method to represent each cohort’s results.

Ethics approval was not required as the evaluation was being conducted in the context of a program and with the goal of improving program quality and delivery. To maintain confidentiality and minimize potential bias in the surveys and pre/post assessments, participants were not required to provide their name or answer open-ended questions. When qualitative responses to open-ended questions were submitted, these were synthesized using frequency mapping (i.e., sorted into common themes for analysis and reporting).

The two end-of-cohort focus groups were recorded with permission from participants over zoom and conducted using standardized, semi-structured facilitation guides. To reduce bias in reporting, focus group facilitators used generic identifiers for each participant name; the zoom recordings were transcribed verbatim and analyzed in NVivo 15 using an inductive, node-based codebook to synthesize recurring themes. To evaluate the mentorship component, the team collected mid-point and end-of-mentorship surveys and facilitated an information group check-in session for mentors to talk about their experiences. The mentor surveys were analyzed descriptively due to the small sample size (*N* = 12 (Y1); *N* = 15 (Y2)).

## Findings

Year 1 to 2 participant findings are organized by themes related to the program’s short-term outcomes: knowledge and skill development, confidence in career pursuits, mentorship, professional networks, real-world application and overall program satisfaction. The results are taken from pre- and post-assessments, end-of-program surveys, and focus groups. Findings from mentor surveys are also reported. Percentages are rounded for reporting purposes.

The participant response rate was strong across both years, with 100% completion of pre-assessments and end-of-program surveys (see Table 3). Post-assessment response rates declined from 92% in year 1 to 75% in year 2, while focus group participation increased from 58% to 75% over the same period. The mentor response rate is average for both mid-point and end-of-mentorship period with between 60% and 67% completion rates.

**Table 3:** Response rates.

PARTICIPANT RESPONSE RATES		
**Evaluation methods**	Year 1 (12 participants)	Year 2 (16 participants)
Pre-assessment	12 (100%)	16 (100%)
Post-assessment	11 (92%)	12 (75%)
End-of-program survey	12 (100%)	16 (100%)
End-of-program focus group	7 (58%)	12 (75%)
**MENTOR RESPONSE RATES**		
**Evaluation methods**	Year 1 (12 mentors)	Year 2 (15^2^ mentors)
Mid-point mentor survey	N/A	10 (67%)
End-of-mentorship survey	8 (67%)	9 (60%)

1. ***Knowledge and skill development***
  Across both cohorts, participants demonstrated strong gains in global health knowledge. Focus group discussions reinforced these shifts (see Table 4). Participants across cohorts described moving from narrow understandings of global health to recognizing its interconnected nature, the influence of local–global disparities and power relations between the Global North and Global South. Year 1 participants described adopting a more “all-encompassing and transcendent” view of global health, while year 2 participants emphasized thinking more “laterally” and paying attention to “all the different factors that come into play on a personal…societal…[and] departmental level.”
  **Table 4:** Participant-reported changes in knowledge and skills.

CONCEPTUAL KNOWLEDGE	YEAR 1^3^		YEAR 2	
	PRE	POST	PRE	POST
Level of knowledge in global health	33%	91%	50%	92%
Level of knowledge in gender equity	33%	91%	81%	92%
Level of knowledge on intersectionality	8%	100%	25%	92%
**Skill application**				
I understand the global health architecture and the forces that influence in-country programs and policies	17%	91%	19%	92%
I am able to conduct gender analysis on specific workplace situations, case studies, or policies	8%	100%	6%	92%
  Across both cohorts, participants demonstrated strong gains in gender equity and intersectional knowledge when applied to institutions and health systems. Focus group findings reinforced these improvements, revealing deepening awareness of systemic gender inequities. Year 1 participants gained “language to something that [we] were not previously able to articulate,” which enabled them to reflect on “gender issues existing in everyday life and in the workplace.” Year 2 participants similarly reported seeing inequities through “a lens of intersectionality” that they are “more cognizant of… in every interaction,” and that hearing others’ experiences affirmed that it was “something that needs to be addressed.”
  Both cohorts described moving from awareness to intentional action. Many reflected on the idea of moving away from seeing gender equity as a checkbox exercise and instead focusing on creating a sense of participatory agency within their workplace.
2. ***Confidence in professional growth***
  Survey results showed solid gains in participant confidence to succeed in global health leadership roles. Agreement with the statement “I am confident I can succeed in a variety of global health leadership positions” rose from 58% to 100% in year 1, and from 75% to 100% in year 2.
  Focus groups further illuminated this shift: Year 1 participants reported moving from a limited understanding of global health as a discipline to seeing themselves as agents of change in the field. Year 2 participants described a shift from seeing themselves solely as “implementers of policies and protocols” to understanding the value of their frontline experience and insight.
3. ***Mentorship***
  The program successfully enabled participants to feel better prepared to mentor emerging leaders. Agreement with the statement “I am confident I can mentor other emerging leaders” increased from 42% to 100% in year 1, and from 69% to 100% in year 2.
  In year 2, confidence in adopting nonhierarchical behaviors rose from 50% to 100%. Across cohorts participants reported improvements in self-reflection, listening, divergent thinking, and in the use of different approaches to mentoring junior colleagues.
  However, survey results indicated a clear, evolving need for improving mentorship training for participants and mentors. In focus groups, participants noted that the mentorship philosophy remained too abstract. A few participants also described challenges due to their mentor’s busy and conflicting schedule, which led some to feel that their mentor was not as interested in building a relationship with them.
  For mentors, while 38% in year 1 and 56% in year 2 found training to be adequate, a significant number requested more support: 100% in year 1 and 44% in year 2. Mentors also requested more opportunities for mentor group sessions to share lessons and to learn varying mentorship modalities. These insights indicate that future program cycles must focus more on enhanced mentor training and relationship-building opportunities. Yet, despite the training gaps, most mentors reported having positive experience with their mentees and with the program in general. They also noted personal and professional benefits, including enhanced listening skills and deeper self-awareness.
4. ***Professional networks***
  Agreement with the statement, “I have strong professional networks that I can tap into to pursue global health leadership positions” increased from 8% to 73% in year 1 and from 50% to 100% in year 2, indicating substantial growth in learners’ perceived access to professional networks across both cohorts.
  Focus groups across both years validated this growth, with participants consistently highlighting their peer-to-peer connection or feeling part of a “sisterhood.” As one participant noted “we now have more people to call on and to exchange ideas… to have a sounding [board]… not just… mentors, but… co-fellows…” The group described leaving the fellowship with a broader and deeper professional network, viewing peers not just as implementation partners but as co-collaborators and problem solvers.
5. ***Real-world application***
  Participants in both years reported feeling more prepared to lead workplace projects addressing gender inequities (see Table 5). Year 1 focus group data indicated that participants gained a deeper understanding of leadership principles and applications, though they identified the need for enhanced leadership content. These insights led to enhancements in the curriculum in year 2, such as providing more leadership theory and self-reflective leadership/journaling sessions. By the end of the second cohort, 80% of participants noted that the program had given them greater self-awareness, improved conflict management and emotional regulation, and permission to adapt their leadership style to specific situations. One participant wrote that the self-reflective/journaling activities “…revealed that authentic leadership isn’t about operating solely from my innate style, but about consciously adapting it.”
  **Table 5:** Participant reported changes in their ability to lead workplace project.

CONFIDENCE AND SKILL APPLICATION	YEAR 1		YEAR 2	
	PRE	POST	PRE	POST
I am confident in my ability to lead gender equity projects that facilitate policy-related change in my workplace.	8%	82%	38%	100%
I can guide decision-making at my organization to promote gender equity.	N/A	100%	38%	92%
  In the year 2 focus group, participants described becoming more self-aware of how they “show up as a leader.” Others spoke about recognizing themselves in different leadership styles and learned about how to apply and adapt their leadership style to different situations and anticipate their effect on others. Finally, participants described improving their ability to set professional boundaries, including learning the value of saying “no.”
6. ***Overall program satisfaction***
  Participants expressed a consistently high level of satisfaction with the fellowship; 100% of participants across both years agreed that they were satisfied with their fellowship experience *(*73% strongly agreed and 27% agreed). In addition, 100% across both years found the leadership project to be useful to their overall leadership journey. Participants stated that they were all likely to implement the project beyond the fellowship.
  In focus groups, participants praised the syllabus and cross-cultural learning between Kenyan and South African health systems, and for bringing together multiple strands of content in a coherent, practical, and useful way. Participants spoke about how the program was well organized and accommodated their busy personal and professional lives. Participants lauded the high level of program organization, noting recordings and flexible timelines that accommodate their busy professional schedules.

### Program strengths

Based on participant and mentor findings, the following summarizes key program strengths:

- ***High participant engagement and satisfaction:*** Participants in both cohorts expressed a high level of satisfaction with the program, specifically highlighting the in-person training, the comprehensive curriculum, their experience with the leadership project, and the professional networks they developed.
- ***Collaborative teaching approach and high-quality facilitators/mentors:*** Faculty and the program coordinator worked together to foster a collaborative teaching environment that played to their strengths by co-teaching sessions, sourcing high-quality guest speakers and mentors, and integrating participants’ knowledge and experiences into the learning.
- ***Responsive and organized program staff:*** Participants noted that program staff responded promptly, gave timely feedback, and supported participants expertly throughout the year.
- ***High growth in participant reported leadership skills and self-awareness:*** Participants reported a deeper understanding of how their personal history, values, attitudes, and behaviors influence their leadership approach. In year 2, the added component of self-reflective leadership sessions and a personal coach drastically improved the program’s ability to influence participant leadership skills and self-awareness.^4^
- ***Strong practical application through the leadership project:*** All participants were very satisfied with their project implementation experience. The project turns theory into real institutional change that produces a tangible workplace impact that the participants were proud of. Key to the project’s success is the support participants obtained from their employers and work colleagues. Several employers have communicated a willingness to continue to support staff involved in the fellowship.
- ***Supportive cross-cultural learning environment:*** A collegial and highly supportive atmosphere prevailed among faculty, participants, and staff, creating a strong sense of community. Participants mentioned they could “laugh together” and bond with their peers in an “equal environment without hierarchies.” Collegiality between the Kenyans, South Africans, and Canadian staff, faculty, and participants was especially evident during the weeklong training and continued until the end of the fellowship.

### Areas for improvement

Based on findings, along with faculty and staff observations, the following areas for improvement were identified:

- ***Mentorship:*** Participants and mentors expressed the need for improved mentorship training. In addition, while most participants had positive experiences, some reported trying to work with mentors with limited availability. The program needs to work on improving training and setting clear expectations ahead of pairings.
- ***Leadership project implementation and sustainability:*** While staff have successfully guided participants in designing feasible workplace projects, without seed funding, projects are not able to maximize longer-term impact.

### Limitations of the evaluation

First, the evaluation findings primarily rely on self-reported participant data collected before, during, and at the end of the fellowship, some of which can be subject to recall bias. Second, the small sample size and the specificity of the Kenyan and South African participants mean that the results are unique to these cohorts and may not be generalizable to a broader group of pan-African women professionals in global health. Third, while the program aims to influence participants’ career roles and the impact of their leadership projects, we have not yet collected data on medium- to long-term outcomes post-fellowship.

Nonetheless, in alignment with Patton’s utilization-focused evaluation methods, the team collected and triangulated participant data at multiple points using various methods (survey data, informal feedback, and focus group), which provided a comprehensive picture of individual and collective changes in knowledge, confidence, and overall growth levels throughout the fellowship [17]. Future evaluation efforts will focus on assessing participant career changes post-graduation, the scaling and sustainability of leadership projects, and institutional learnings related to the tri-academic partnership within the broader AHC.

## Discussion

A leadership capacity-building program can contribute to advancing gender equality, but relying on it as a primary strategy has inherent limitations in impacting societal norms. Gender, as a socially constructed system of differentiation, does not operate in a vacuum and intersects with other oppressive systems including racism and ableism in geopolitical and sociocultural contexts. System-level changes (e.g., laws to promote equal pay; inclusive policies that increase women’s representation in leadership roles) are also required to accelerate gender transformation. Nonetheless, a program like the WGHLF can contribute to increasing gender awareness and gender-sensitive practices at individual and organizational levels. For example, participants reported notable increases in gender awareness and intention to act, and several of their leadership projects resulted in policy changes, such as a new workplace breastfeeding policy and training protocols to treat survivors of gender-based violence.

Power asymmetries inevitably exist within a collaborative involving Global North and Global South higher education institutions. To mitigate these imbalances, the three partners established a governance mechanism with clearly articulated roles and contributions for each member. This has helped to ensure ongoing collaboration and transparency in decision-making about program design, content delivery, and implementation. For the UofT’s Centre for Global Health, staff intentionally work on active listening and strive to offer guidance in a way that maintains the African partners’ sense of agency. Shared values of mutual learning and reciprocity between partners are reinforced through ongoing communication (online and in person), which has led to ongoing buy-in, shared resource allocation, implementation, and evaluation. Finally, integrating African case examples and perspectives has continuously grounded program discussions in the partner’s lived experiences.

### Implications for initiatives to advance women’s leadership in global health in LMICs

The study results have broader implications for advancing gender equity through global health leadership programming by demonstrating how a co-created capacity-building fellowship program between academic institutions in the Global North and South can strengthen women’s leadership pipelines and help redistribute leadership authority in LMIC contexts [18]. The program has achieved its short-term outcomes while remaining nimble and responsive to changing needs. Given growing demand for this program, consideration is now being given to different pathways to scaling for impact—from scaling up (integrate program into systems) to scaling out (expanding participant numbers or academic partners involved) to scaling deep (continuous improvement of program quality to create lasting impact) [19]. To date several scaling deep strategies are considered to help preserve program quality, integrity, and safety in learning that comes with a small cohort. Program enhancements such as additional leadership theory, leadership self-reflection/journal questions, and one-on-one coaching (in addition to group coaching) were integrated into the year 2 curriculum based on feedback from the first cohort. In year 3, mentorship expectations and training will be augmented. While several leadership projects have successfully launched, plans are in place to provide seed funding to further scale their impact. To support an increasing number of well-qualified candidates, academic partners and fellowship alumni are co-creating a community of practice to include a broader number of women leaders beyond those who are admitted into fellowship. Partners are intentionally considering succession planning and sustainability beyond funding for the AHC by involving alumni in program leadership and content delivery.

## Conclusion

The global health systems field is weakened by the loss of female talent, which impacts the achievement of several Sustainable Development Goals (SDG) including, including SDG 3 (Universal Health Coverage) and SDG 5 (Gender Equality) [2, 3, 9]. To address this challenge, the WGHLF supports early-to-mid-career women in Kenya and South Africa to advance their leadership capacity to push forward gender equity practices in global health spaces. Participants reported significant improvements in their confidence as future leaders and in their knowledge of global health, gender equity, leadership, and mentorship. This type of collaborative fellowship training, engaging global north and global south institutions, can serve as a model for others seeking to prepare women for higher-level leadership positions, crucially needed to advance gender equity and local health systems.

## Notes

Two participants worked together to launch one project at one entity, hence the total of 27 projects for 28 participants.

One mentor worked with two participants, hence 15 mentors for 16 participants in year 2.

Number of participants: Y1 = 12; Y2 = 16. Percentages represented in the table are based on the number of participants in each year.

This program strength applies only to year 2. It was captured from the end of year focus group due to key changes made at the end of year 1.

## Additional File

The additional file for this article can be found as follows:

Supplementary Figure 1. Women in Global Health Leadership Fellowship – Logic Model.

## References

[r1] UN Women. Facts and figures: Women’s leadership and political participation. Published 2026. Accessed March 17, 2026. https://www.unwomen.org/en/articles/facts-and-figures/facts-and-figures-womens-leadership-and-political-participation.

[r2] Newman C, Chama PK, Mugisha M, et al. Reasons behind current gender imbalances in senior global health roles and the practice and policy changes that can catalyze organizational change. Glob Health Epidemiol Genom. 2017;2:e19. https://pubmed.ncbi.nlm.nih.gov/29868225/. 29868225 10.1017/gheg.2017.11 PMC5870424

[r3] WHO Global Health Workforce Network Gender Equity Hub. Delivered by Women, Led by Men: A Gender and Equity Analysis of the Global Health and Social Workforce. World Health Organization; 2019. https://www.who.int/publications/i/item/9789241515467.

[r4] Dhatt R, Theobald S, Buzuzi S, et al. The role of women’s leadership and gender equity in leadership and health system strengthening. Glob Health Epidemiol Genom. 2017;2:e8. https://pmc.ncbi.nlm.nih.gov/articles/PMC5870471/. 29868219 10.1017/gheg.2016.22 PMC5870471

[r5] Global Health 50/50. Gaining global ground? analysis of the gender-related policies and practices of 201 global organizations active in health. Published 2024. https://global5050.org/wp-content/themes/global-health/reports/2024/media/Gaining%20Ground_GH5050%202024%20Report_Online.pdf.

[r6] Liu E, Iwelunmor J, Gabagaya G, et al. Women’s global health leadership in LMICs. Lancet Glob Health. 2019;7(9):e1172–e1173. https://www.thelancet.com/journals/langlo/article/PIIS2214-109X(19)30308-0/fulltext. 31401998 10.1016/S2214-109X(19)30308-0

[r7] Downs JA, Reif LK, Hokororo A. et al. Increasing women in leadership in global health. Acad Med. 2014;89(7):1103–1107. doi:10.1097/acm.0000000000000369. 24918761 PMC4167801

[r8] Alla F, Cambon L, Ridde V. Population Health Intervention Research: Concepts, Methods, Applications. IRD Éditions; 2023. https://www.editions.ird.fr/produit/699/9782709930048/.

[r9] Langer A, Meleis A, Knaul FM, et al. Women and health: The key for sustainable development. Lancet. 2015;386(9999):1165–1210. doi:10.1016/S0140-6736(15)60497-4. 26051370

[r10] Singh S, et al. Protecting global sexual and reproductive health and rights in the face of retrograde U.S. policies and positions. Lancet. 2025;405(10490):1650–1653. https://www.thelancet.com/journals/lancet/article/PIIS0140-6736(25)00618-X/abstract. 40188843 10.1016/S0140-6736(25)00618-X

[r11] Global Health Council. Statement from the Global Health Council on the mass termination of USAID and State Department grants. Published 2025. Accessed March 11, 2026. https://globalhealth.org/media/statement-from-global-health-council-on-the-mass-termination-of-usaid-and-state-department-grants/.

[r12] Carnegie Endowment for International Peace. The New Global Struggle Over Gender, Rights, and Family Values. Published 2025. Accessed March 13, 2026. https://carnegieendowment.org/research/2025/06/the-new-global-struggle-over-gender-rights-and-family-values?lang=en.

[r13] Africa Health Collaborative. Home. Published 2024. Accessed June 3, 2026. https://africahealthcollaborative.org/.

[r14] Kenyatta University Teaching, Referral and Research Hospital. Tumaini SGBV clinic officially launched at KUTRRH. Published 2026. Accessed April 3, 2026. https://www.kutrrh.go.ke/tumaini-sgbv-clinic-officially-launched-at-kutrrh/.

[r15] Gillis C. Khayelitsha gets a men’s health clinic. Elitsha. Published 2024. Accessed on April 3, 2026. https://elitshanews.org.za/2024/09/26/khayelitsha-gets-a-mens-health-clinic/.

[r16] Ross McGregor K, Jacobs K. Khanyisa Jacobs: Promoting men’s health in Khayelitsha. Cent Glob Health Newsl. 2024.

[r17] Patton MQ, Campbell-Patton CE. Utilization-Focused Evaluation. 5th ed. Sage Publications; 2021.

[r18] Pai M, et al. Shifting power in global health will require leadership by the Global South and allyship by the Global North. Lancet. 2024;404(10464):1711–1713. doi:10.1016/s0140-6736(24)02323-7. 39491869

[r19] McLean R, Gargani J. Scaling Impact: Innovation for the Public Good. International Development Research Centre; 2019.

