# Supplementary figures and images for: Advancing Women’s Global Health Leadership: Lessons from a Tripartite Model
Source: Ann Glob Health. 2026 Jul 8;92(1):63. doi: 10.5334/aogh.5285 (PMC13353093; doi:10.5334/aogh.5285)

## Supplementary Figure

### Women in Global Health Leadership Fellowship – Logic Model

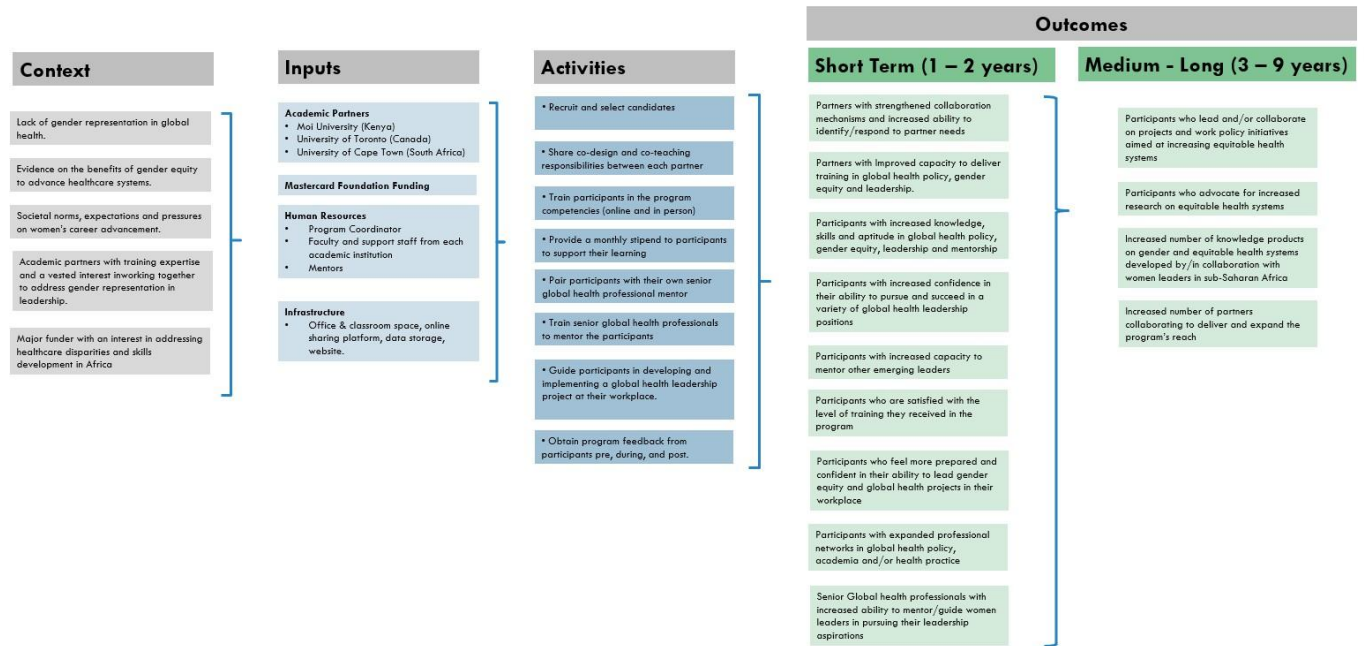

Supplement: Supplementary Figure 1. — Women in Global Health Leadership Fellowship – Logic Model. [file agh-92-1-5285-s1.pdf]
